# Towards an Effective Patient Health Engagement System Using Cloud-Based Text Messaging Technology

**DOI:** 10.1109/JTEHM.2018.2868358

**Published:** 2019-11-12

**Authors:** Chih-Wen Cheng, Clark R. Brown, Janani Venugopalan, May D. Wang

**Affiliations:** 1 The Wallace H Coulter Department of Biomedical EngineeringGeorgia Institute of Technology 1372 AtlantaGA30332USA; 2 Children’s Healthcare of AtlantaAtlantaGA30322USA

**Keywords:** Cloud computing, patient engagement, self-reported outcomes, sickle cell disease, text messaging

## Abstract

Patient and health provider interaction via text messaging (TM) has become an accepted form of communication, often favored by adolescents and young adults. While integration of TM in disease management has aided health interventions and behavior modifications, broader adoption is hindered by expense, fixed reporting schedules, and monotonic communication. A low-cost, flexible TM reporting system (REMOTES) was developed using inexpensive cloud-based services with features of two-way communication, personalized reporting scheduling, and scalable and secured data storage. REMOTES is a template-based reporting tool adaptable to a wide-range of complexity in response formats. In a pilot study, 27 adolescents with sickle cell disease participated to assess feasibility of REMOTES in both inpatient and outpatient settings. Subject compliance with at least one daily self-report pain query was 94.9% (112/118) during inpatient and 91.1% (327/359) during outpatient, with an overall accuracy of 99.2% (970/978). With use of a more complex 8-item questionnaire, 30% (7/21) inpatient and 66.6% (36/54) outpatient responses were reported with 98.1% (51/52) reporting accuracy. All participants expressed high pre-trial expectation (88%) and post-trial satisfaction (89%). The study suggests that cloud-based text messaging is feasible and an easy-of-use solution for low-cost and personalized patient engagement.

## Graphical abstract

We present a clinical study of pain management and provider-patient communication using text messaging. The study suggests that cloud-based text messaging is a feasible low-cost and personalized patient engagement strategy.

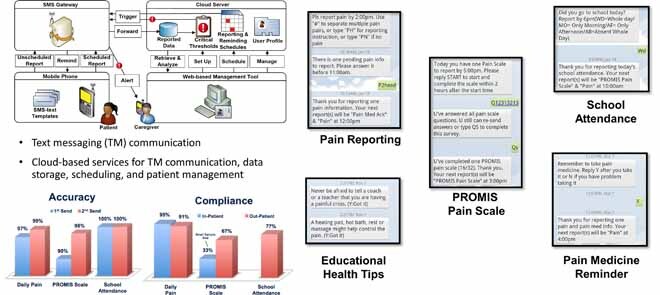

## I. Introduction

Patient engagement is an activity that involves a series of proactive and interactive actions between provider and patient. Active patient engagement is paramount in effective health care, especially in chronic conditions, which are long term, variable, and often degenerative [1]. Direct collection of patient-reported outcomes (PROs) is essential for effective patient engagement. Studies have shown that PROs are beneficial over clinician-reported outcomes by facilitating the efficiency and accuracy of patient-provider communication via real-time symptom tracking instead of manual extracting from medical records in a sparse time points of clinical visits [2], [3]. Paper-based scales are the most typical data collection instruments of PROs, such as ChronoSheet in bipolar disorder [4] and McGill pain questionnaire in chronic pain [5]. However, one of the challenges of paper-based PRO scales is poor compliance for real-time reporting, leading to incomplete or biased recall of events. Previous comparison studies using electronic and paper PROs demonstrated that subjects overwhelmingly fail to complete paper PROs according to protocol (e.g., retrospectively filling in multiple entries at one time; subject failure to return paper diaries). Reported compliance of paper PROs ranged from 11 – 56%, while the compliance of electronic PROs ranged from 83–89% [6]–[8].

In addition, from a provider’s point of view, processing paper-based document is expensive and laborious with a higher risk of human-errors. Thus, modern communication technologies offer promise by enhancing the quality and efficiency of tracking real-time PROs. Among these technologies, mobile phone text messaging is considered a favored alternative [9] due to wide spread use and low cost.

According to the CTIA-The Wireless Association, as of the end of 2012, the number of mobile phone subscribers reached 326.4 million with 102% penetration (i.e., # of active units divided by the total U.S. population) [10], exceeding the percentage of population with computer access. Another study in 2017 also rated text messaging as the top choice for medical alerts and reminders [11]. Among all cell phone activities, text messaging (TM), also known as the short messaging service (SMS), is the most prevalent, both in overall likelihood and frequency [12]. Studies have demonstrated that TM can be an effective PRO collection technology for health interventions [9], [13], [14], clinical management [15]–[18], and health-related behavior modification [19], [20]. In addition, TM extends the capacity for monitoring outpatient disease complications (e.g., frequency and duration/resolution of painful episodes), potentially reducing hospitalizations.

Several features make TM one of the ideal technologies for engagement in self-care. First, because TM is a basic function in every cell phone, providers do not need to develop independent applications on devices. Second, patients can receive and respond to TMs anytime using readily available 2G (e.g., GSM) network and without depending on 3G/4G or wireless networks. Third, TM is cheaper (e.g., unlimited for $10–$15 USD/month) and unobtrusive compared to telephone interviews. Fourth, in terms of PRO collection, TM is conducive for several short queries in a single day. Finally, in terms of longitudinal data collection, TM provides sustainable usage no matter how many times the back-end system is updated by administrator or the mobile device is changed by a user. In addition, the targeted population for SCD is from mainly lower income groups where the issues were on access to smart phones and internet on the phones. Also there were concerns from parents regarding privacy and safety on the use of smart phones for the young adolescents in school. As a result we chose TM as the design choice for this application after discussion with care providers and focus groups.

TM systems in healthcare can be classified into two categories based on the type of communication: one-way reminding and two-way reporting. One-way TM systems are mainly designed to send standardized messages for medication dose scheduling and specific preferences. For example, Strandbygaard *et. al*. used TM reminders to improve asthma treatment and suggested that daily TM reminders could significantly increase adherence to anti-asthmatic medication [21]. Two-way messages are designed for self-report followed by automated feedback. Shapiro *et. al.* demonstrated the feasibility of two-way TM for monitoring physical activity as well as consumption of sugar-sweetened beverages and screen time in children. Subject’s self-report generated an automated TM positive feedback response to re-enforce good behavior [22].

However, several challenges have hindered TM from been widely adopted in tracking PROs. First, a majority of TM reporting systems relied on free-texts read by caregivers, which still causes extra human effort. Second, conventional TM systems often lacked the capability of an automated context-appropriate reply for behavior modification, necessitating providers to manually input responses. As the number of users increase, this process becomes more labor intensive and slower. Third, conventional systems lacked error-proofing with real-time correction; thus, responses were recorded regardless of accuracy. This problem has been especially problematic for younger adolescents or elder populations. Fourth, current TM-based PRO systems are restricted to a fixed reporting schedule for all patients, making it difficult to tailor a schedule according to an individual’s daily activity. Fifth, common format used in past system is restricted to simple responses (such as typing Y (Yes) or N (No)), limiting measurement of complex health outcomes. Finally, TM reporting system do not have automatic provider notifications based on personalized health condition thresholds.

To address the aforementioned challenges, in this study we present a cloud-based two-way text messaging system, nicknamed **REMOTES** (**RE**porting and **MO**nitoring **TE**lemedicine **S**ystem). REMOTES is built on highly scalable and interactive cloud services. It allows providers to establish personalized reporting schedules. TM prompts and interventions are generated and delivered automatically without the need for manual typing. The TM communication is accomplished by utilizing an easy-to-understand reporting template that is capable of complex health assessments. The system can also trigger automatic alarms to providers based on each personalized health condition thresholds. System design and implementation is provided in Section II. Results from a feasibility case study with adolescents with SCD are in Section III, followed by conclusion and directions are in Section IV.

## II. Methods

### A. Use Case and System Design

The architecture of REMOTES is developed using open-source and cloud-based services operated by Google, Inc. A Google App Engine (GAE) platform acts as a central platform incorporating with other cloud services, including Google Voice, Google Drive, and Google Calendar, for different purposes. The GAE service has a web-based interface allowing providers to perform online patient management. The cost-effective framework can handle 30 users in $4/week. Key use cases and their corresponding cloud services are listed in Table 1 and described in the following of this section.

**TABLE 1:** Use Cases and Corresponding Cloud Services

Cloud Service	Related Use Cases
^*^Google App Engine	Patient management Security protection
Google Calendar	Report scheduling Security protection
Google Drive	Message pool storage Report formatting Health Tips Security protection
Google Voice	Text messaging communication Security protection

^*^ In addition to patient management, Google App Engine is the central platform controlling all other use cases.

#### 1) Patient Management

We use the Google Web Toolkit (GWT) to develop an online patient management tool that can access the central GAE platform. Providers can use this online tool to subscribe new patients and specify personalized health condition thresholds. A threshold is a numerical number that triggers alarms to corresponding providers when a patient reports a score above the threshold. For example, if a pain threshold for a patient is 5, a pain score of 6 triggers critical alarms to the patient’s associated providers for just-in-time follow-ups.

#### 2) Report Scheduling

In REMOTES, a patient has his/her own reporting schedule, which is set by providers, on Google Calendar. A calendar can consist of a series of one-time or repetitive events. Whenever the event occurs, the system prompts a corresponding message to the associated patient(s). Providers perform the scheduling through a common calendar interface to minimize additional learning. In addition, providers can share calendars with patients and their families (with or without the privilege of editing) so that the reporting schedule can fit their daily routines.

#### 3) Message Pool Storage

In order to prevent sending repetitive messages to patients, which makes the reporting become tedious, the messages sent to patients are fetched from an event message pools. A message pool allows different expressions of a critical concept. The message pools are stored in spreadsheets hosted on the Google Drive (formally Google Docs) service. Care provider can access the Google Drive service to edit messages when needed. The message pools are sharable so that providers can improve message content together. For example, a physician can share the message pool with a psychiatric to improve messages related to mental health.

#### 4) Text Messaging Communication

The system sends and receives TMs via a unique phone number provided by the Google Voice service. Upon receipt of a response from a patient, the Google Voice service relays the message to the GAE platform where the message is processed. If the response is invalid, REMOTES triggers an instructional message, which contains an example in valid format, and requests the patient to repeat the response. Otherwise, the system stores the response and, if necessary, sends an intervention and/or the next question.

#### 5) Report Formatting

A majority of conventional TM reporting systems communicate via free-text and do not impose message formatting. Manually processing unformatted messages requires effort from medical staff, and may result in human interpretation errors. We introduce a simple response format that can be automatically processed by computers and is capable of a wide coverage of reporting styles. The formatting instruction is embedded in the messages sent to the patient, alleviating a need to remember response formats. This schema gives providers the flexibility to define templates. All templates are stored on the Google Drive.

#### 6) Health Tips

In addition to the self-reporting via two-way text messaging, REMOTES is also capable of sending health tips via one-way messages that do not require patients to respond. Health tips provide educational information that encourages patients to learn about how to maintain their health and prevent possible conditions. Health tip messages are generated by providers and stored in message pools on Google Drive. Providers can help patients schedule when they want to receive health tips on the calendar. When a health tip event occurs, the REMOTES system randomly selects and sends a message form the pool.

#### 7) Reports Storage and Retrieval

In addition to message pools, the Google Drive service stores patient reports. When a response is received from a patient and is valid, it is stored in a patient–specific spreadsheet. Providers can log in to download data in the file formats used by common data analysis software. Google Drive service also stores important information regarding patient interaction with the REMOTES system, including (1) number of messages sent to patients, (2) number of messages received, and (3) other reporting statistics (e.g., on-time reporting rate, reporting correctness, and average response time).

#### 8) Security Protection

Security and privacy concerns are significant for cloud computing systems, creating several challenges for use in the healthcare domain [23]. Our system design strategy was to remain HIPPA-compliant and secure personally identifiable information. None of the services linked to any medical record systems, and none of stored data on Google Drive could be mapped to a patient medical record. A specially created google voice number will be used for each patient and the mapping will be stored in the HIPPAA complaint systems at hospitals. All generated data reports contained only de-identified and aggregated data. Access to all Google services was controlled by a unique account with a high-strength password that was changed every three months at a minimum and shared only with authorized personnel. A study contact email address was given to patients to report system errors or unexpected behavior. Even though Google’s cloud services provided high level of data protection, our data on the Google Docs was automatically backed up twice a week using secure Internet protocol, encrypted, and then archived in the password-protected server.

## III. Clinical Study Design

Sickle Cell Disease (SCD) is the most prevalent hereditary disease, particularly among people with an African descent. Despite the advancement in medicine, SCD remains as a major health problem, worldwide. SCD is characterized by episodic vaso-occlusive crisis (VOC) in multiple body sites (e.g., abdominal and bone), often leading to damage of body organs. VOC can cause severe pain, which is the most common reason for hospitalization.

As an inheritable disease, SCD affects a patient’s life as early as 9 months of age. A North American study estimated that 10 to 19 year-old SCD teenagers have 8-fold probability of death, as compared to age- and race-matched controls [24]. Effective patient engagement for adolescents with SCD requires frequent medical monitoring, including tracking the duration and severity of VOC. Conventional monitoring systems are typically paper-based or web-based diaries requiring patients to carry forms or notebooks, which are impractical, and prone to sporadic use by adolescent patients [25]. In order to improve the patient engagement in pediatric SCD, in this case study, we have adopted REMOTES to deliver two-way text messages for effective self-reporting. We aim to demonstrate the feasibility of the REMOTES by high compliance rate, reporting accuracy, and response efficiency in both inpatient and outpatient environments.

### A. Clinical Setting

A feasibility study was conducted in the Clinical Research Organization of the AFLAC Cancer Center & Blood Disorders Service in Children’s Healthcare of Atlanta (CHOA), Atlanta, Georgia. The AFLAC Cancer and Blood Disorders service coordinates the care of one of the nation’s largest comprehensive sickle cell centers actively providing over 1,600 pediatric patients. For 33 years, the service has supported clinical and research efforts by providing a variety education and advocacy services.

The present study was approved by the Institutional Ethics Review Board and required all enrolled subjects to understand and provide written assent and all parents/guardians to provide written consent.

### B. Measures

Measures in this study included daily pains, daily school attendances, and weekly scores of a pain interference scale.

#### 1) Daily Pain

Pain crisis is the most common symptom in SCD, which requires frequent monitoring. In our study, pain episodes, including intensities and locations, were queried daily from teen participants. The pain template consists of a header ‘**P**’ followed by a series of intensities and locations in pairs. The sensory pain intensity was reported on a 1–10 numerical scale where “10” represents the worst imaginable pain. Eight pain locations were included for most common pain sites in SCD [26], including ‘HEAD’, ‘CHEST’, ‘BELLY’, ‘BACK’, ‘UL’ (upper left extremity), ‘UR’ (upper right extremity), ‘LL’ (lower left extremity), and ‘LR’ (lower right extremity). For example, a message ‘P2CHEST&3BELLY’ represented two location of pain: chest pain with intensity two and abdominal pain with intensity three. A simple response PN was used if there was no pain to report.

#### 2) Daily School Attendance

Evidence has shown that lower academic performance is highly associated with the increase in the number of pain episodes and their severity in children and adolescents with SCD [27]. However, conventional self- or parent-reported school attendance inherently suffers from misreporting and non-compliance issues, which is of particular significance with adolescent populations. Therefore, school attendance was another measure that requires adolescent to report every evening. The report requires no header but one possible 2-character response from four options regarding the attendance, including ‘WD’ (Attended Whole Day), ‘AM’ (Absent Only Morning), ‘AF’ (Absent Only Afternoon), and ‘AW’ (Absent Whole Day).

#### 3) Weekly PROMIS Pediatric Pain Interface Scale

The National Institute of Health (NIH) Patient Reported Outcomes Measurement Information System (PROMIS) initiative is developing self-reported measures, which cover a wide variety of health domains. The PROMIS Pediatric Cooperative Group focuses on the development of pediatric self-report scales for ages 8 through 17 years. All items of PROMIS pediatric scales use a seven-day recall period. Thus we included the PROMIS Pediatric Pain Interference short form for a weekly-based assessment on pain interference [28]. The scores for the eight PROMIS questions were reported using a series of numbers ranging from zero to four, such as ‘01324101’.

#### 4) Health Tips

The system sent age- and disease-appropriate health tips to participants once a day without requiring a response. A health tips was randomly selected from a pool of 54 messages in 11 subsets relating to critical topics, such as pain management and transition to adult care. These tips provide important knowledge that encourages adolescents with SCD to learn about how to manage their diseases.

### C. Participant Enrollment and Training

We enrolled 27 teenagers with SCD who were hospitalized for uncomplicated VOC painful crisis. All patients had a 10 cm visual analogue scale pain score ≥ 4.5/10 at the time of enrollment. Potential subjects were excluded based on comorbid conditions associated with complicated VOC (e.g., evidence of acute chest syndrome, ongoing treatment with monthly erythrocyte transfusion), or any physical impairment, including prior history of stroke that would limit ability to perform text messaging. The average age of enrolled subjects was 14.6 (10–18) years with 13 (48.1%) males. Nine (33.3%) agreed to finish only in inpatient protocol, four (14.8%) agreed only outpatient protocol, and all other 14 (51.9%) agreed to complete both the two protocols. The mean length of inpatient study was 5.1 (2–16) days with total 118 trial days from 23 patients. The mean outpatient length was 19.9 (8–32) days with total 359 trial days from 18 patients.

The training of text messaging monitoring was initiated during hospitalization for all subjects (even for those who only agreed to finish the outpatient protocol). After consent, study coordinators provided a training section to introduce the REMOTES system and demonstrated how to perform effective reporting. Afterwards, the coordinators tracked reporting performance and monitored captured data. Pre-trial expectation and post-trial satisfaction surveys were performed on a patient’s first day and last day, respectively.

### D. Report Scheduling

**FIGURE 1.**
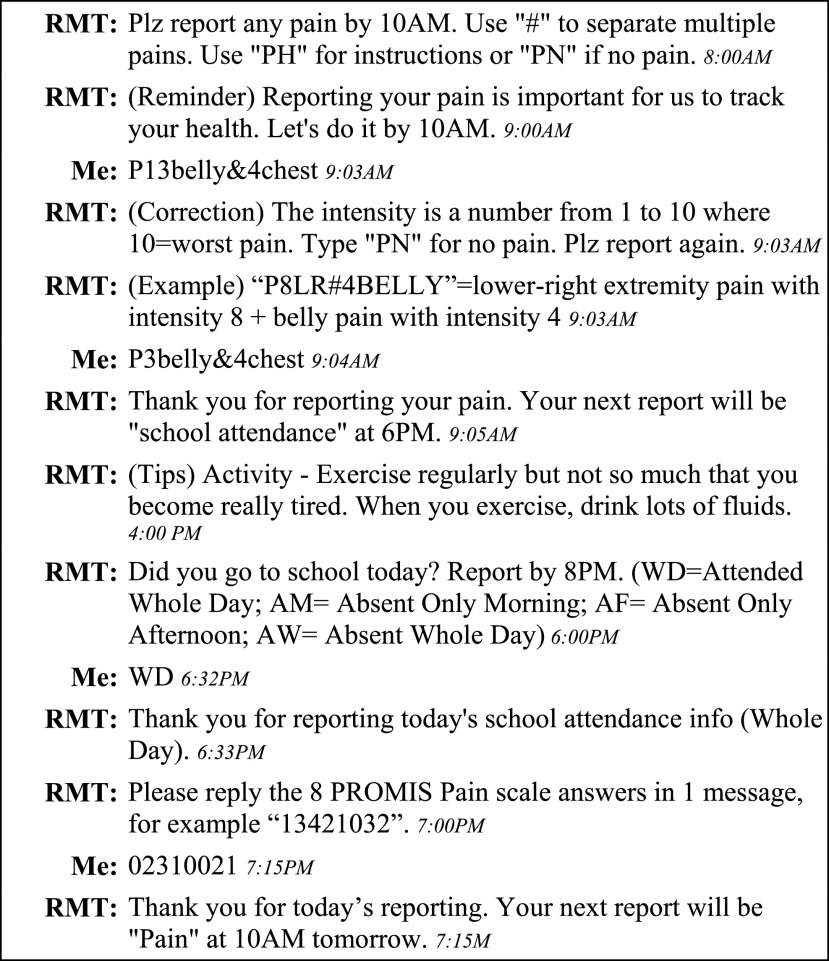
An example of REMOTE conservation between the system (RMT) and an adolescent (Me). The conversation includes a pain query (8:00AM) followed by instruction on format correction, a daily health tip (4:00PM), and a school attendance query (6:00PM). This conversation also includes the weekly 8-question PROMIS survey (7:00PM). The use of reminder message is also demonstrated.

At the end of a training section, the study coordinator created a reporting schedule, or assigned an existing schedule, to the patient. Patients in inpatient protocol had three chances (between 7AM and 7PM) to report their daily pains. Patients in outpatient protocol also had three chances but the schedules were personalized to their daily activities (e.g., before school, after school, and before bedtime). Although all inpatients and outpatients were required to report daily pain at least once a day, multiple reports were encouraged. Report of school attendance was required only in outpatient protocol and occurred around dinnertime. Each pain or school attendance query was allowed a time window for completion (i.e., the event length on the calendar). Within a time window, reminder messages were sent if no response was received. Finally, the PROMIS pain scale was required once every weekend. An example of two-way reporting is illustrated in Fig. 1.

## IV. Results

### A. Daily Pain

The summary of results is listed in Table 2. In total 118 inpatient pain query days from 23 patients, 112 out of the days had pain records resulting in a compliance rate of 94.9%. The system received 383 inpatient pain messages among which 378 (98.7%) were valid following the reporting template. Eight (2.1%) out of the 378 messages were initially invalid, but corrected in real-time via error-proofing message. Three hundred and thirty (87.3%) pain messages were received on time (i.e., reported within allowed time windows). The average response time of on-time pain messages was 36.2 minutes. As for outpatients, 327 out of 359 pain query days had pain records, resulting in a compliance rate of 91.1%. The system received 595 outpatient pain messages, and 592 (99.5%) of the responses were valid. Fourteen (2.4%) out of the 592 responses initially used an invalid format, but eventually corrected via error-proofing messages. Four hundred and twenty-three (71.1%) of the pain messages were received on time with average reporting time of 43.5 minutes.

**TABLE 2:** Compliance and Correctness With Txt Reports

Variable	In-Patient Protocol	Out-Patient Protocol
Pain (daily query)		
^*^No. of Patients	23	18
Query days	118	359
Average patient days	5.1 (2–16)	19.9 (8–32)
Days reported	112	327
Compliance	94.9%	91.1%
Response in valid format	98.7%	99.5%
On time response (response time in minutes)	87.3% (36.2)	71.1% (43.5)
School attendance (daily query)		
No. of Patients	-	6
Query days	-	90
Days reported	-	69
Compliance	-	76.7%
Response in valid format	-	100%
On time response (response time in minutes)	-	81.2% (34.1)
PROMIS Scale (weekly query)		
No. of Patients	23	18
Query weeks	21	54
Weeks reported	7	36
Compliance	33.3%	66.7%
Response in valid format	100%	97.8%

^*^ Fourteen subjects completed both protocol

### B. School Attendance

Outpatients had chances to report daily school attendance. Sixty-nine out of 90 (76.7%) query days were acknowledged. All (100%) school attendance messages were reported correctly. The on time reporting rate was 81.2% (56/69) with average reporting time of 34.1 minutes. However, the number of school days for reporting varied between participants. A few participants and their parents opted not to report school attendance. Also, school calendars varied by for the different school systems, limiting the ability of the Study coordinator to account for some official school breaks.

### C. PROMIS Pediatric Pain Interface Scale

The PROMIS Pediatric Pain Interface scale was prompted once every week in both inpatient and outpatient protocols. Because patients had flexibility to report during the period from Friday evening to Sunday evening, the response time was not recorded. In addition, patients were allowed to resend PROMIS messages to change answers. There were totally 21 inpatient query weeks from 23 inpatient children. Seven (33.3%) queries were acknowledged. All (100%) of the seven messages were in valid format.

The outpatients had 54 PROMIS query weeks in which 36 (66.7%) queries were responded. The system received 45 PROMIS messages among which 44 (97.8%) were reported validly following the PROMIS template. Four out of the 45 messages were answered correctly after the error-proofing instruction.

### D. Expectation and Satisfaction

We utilized two surveys to assess the system’s pre-trial expectation and post-trial satisfaction. Each survey consists of 15 questions in three main groups: ease of use (e.g., “It would be easy for me to communicate to my nurse and doctor by using REMOTES”), useful for disease management (e.g., “Using REMOTES is a smart way of helping me manage my SCD”), and useful for family (e.g., “Using REMOTES would be valuable to my family”). Each question was answered from 0 (strongly disagree) to 6 (strongly agree).

**FIGURE 2.**
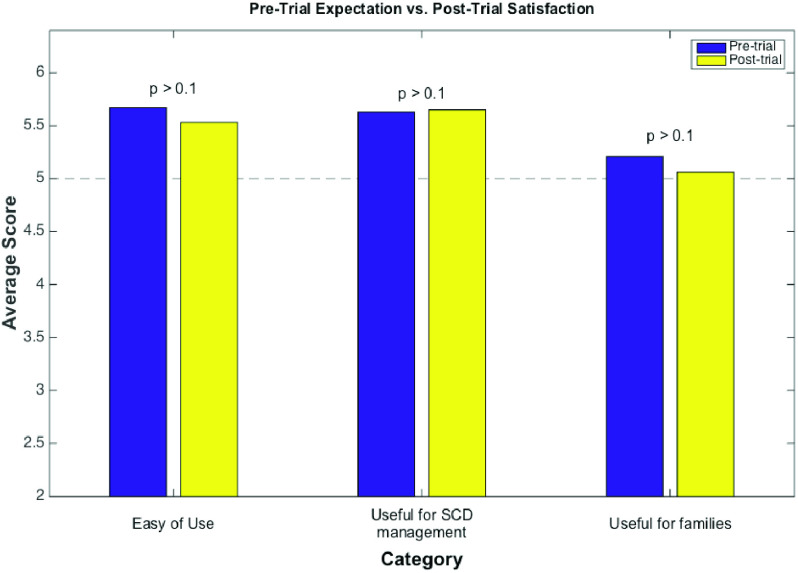
Comparison of survey scores of pre-trial expectation and post-trial satisfaction.

Totally 19 pre-trial questionnaires and 19 post-trial questionnaires were returned. As shown in Fig. 2, patients had high (average score > 5) expectation in all three categories and still held high satisfaction at study exit. Patients were provided an opportunity to give written comments on the experience of the REMOTES system. Based on written comments on the study questionnaires, most of the subjects welcomed the REMOTES system to manage the SCD, especially during pain crisis, and would recommend the system to other people with SCD. For example, one subject commented, “Loved it! I wish I could use it (at) home in the future. This mainly works with people who use cell phones and those that are outpatients.”

## V. Discussion

This study demonstrates that PROs are effective tools for monitoring recurrent pain in children with sickle cell disease. As far as we are aware, this is the first study demonstrating the clinical feasibility of a low-cost electronic PRO, using cloud-based and two-way text messaging to promote self-care in adolescents with pain. This initial pilot study using REMOTES shows an equal or greater compliance, in comparison to other studies using PDAs [6], [29], Smartphones [7], [30], [31], or web-based tools [32]. We propose that the level of compliance and satisfaction with the REMOTES system is due to, in part, a system design that allows participants’ ability to merge Study reporting with their personal text messaging and to personalize reporting schedules. In general, the REMOTES system provides a significant improvement to conventional paper-based reporting [33] and to text messaging adaptations in other healthcare settings.

Reporting accuracy is an indicator of the system’s easy-of-use. Our results showed that adolescents with SCD could perform text messaging reporting with very high accuracy (i.e., all >97%) using varied formats to report school attendance (i.e., two-character format), to report pain (i.e., combining descriptors of intensities and locations), and to respond to a multi-item questionnaire (i.e., answering eight questions in one message). The accuracy was even improved more by using descriptive instructions on valid format.

Finally, the survey indicated that most patients had high pre-trial expectation and still hold highly positive attitude after using the REMOTES. However, there are some unresolved issues:

(1) Outpatients had lower (71.1%) on-time reporting rate. Differences in report time may reflect changes in subject schedule and phone accessibility in the outpatient setting.
(2) A lower level of compliance was observed with weekly PROMIS questionnaire, when compared to daily pain reporting. Studies have suggested that that length and survey complexity have a negative effect on self-report using electronic diaries. While our system designed reduced complexity, additional format adjustment or subject incentives may be required attempting multi-item surveys. It is less clear why compliance with daily school attendance reporting was significantly lower than daily pain reports. We speculate that the perceived relevance and/or the consequences of school absenteeism may have negatively influenced subject self-report. Additional discussion at study entry that enhances the participant’s understanding of the query’s importance may improve compliance.
(3) The pre- and post- surveys did not show improvement. The reason likely reflects a survey design that underestimated baseline perceptions about the system’s quality and usability. The adolescent population had a high of familiarity with TM-based technology prior to the study through continuous and ongoing use. Future studies will need to account for the subjects’ technical sophistication as well as improve satisfaction assessment by applying advanced technology acceptance models [34].

## VI. Conclusion

Conventional paper- or web-based monitoring systems introduce error and data lapses that may result in inaccurate clinical trial results, dissatisfaction with health care, preventable complications, or hospital readmissions. We present a novel system using two-way text messaging technology to achieve a cost-effective, easy-to-use, unobtrusive, and timely health monitoring channel. Clinical application to adolescents with sickle cell disease demonstrates feasibility and acceptability of the REMOTES system to promote patient health engagement on a frequent basis. We hope this study can provide a good example to system developers and care providers who want to develop a self-reporting system using text-messaging technology.
